# Supplementary material for: Optimized feature gains explain and predict successes and failures of human selective listening
Source: Nat Hum Behav. 2026 Mar 13;10(5):937–59. doi: 10.1038/s41562-026-02414-7 (PMC13192276; doi:10.1038/s41562-026-02414-7)
Supplement: Supplementary file 1 — Supplementary Figs. 1–5. [file 41562_2026_2414_MOESM1_ESM.pdf]

nature human behaviour

Supplementary information https://doi.org/10.1038/s41562-026-02414-7

Optimized feature gains explain and predict successes and failures of human selective listening

In the format provided by the authors and unedited

Supplementary Figures for Griffith, Hess & McDermott

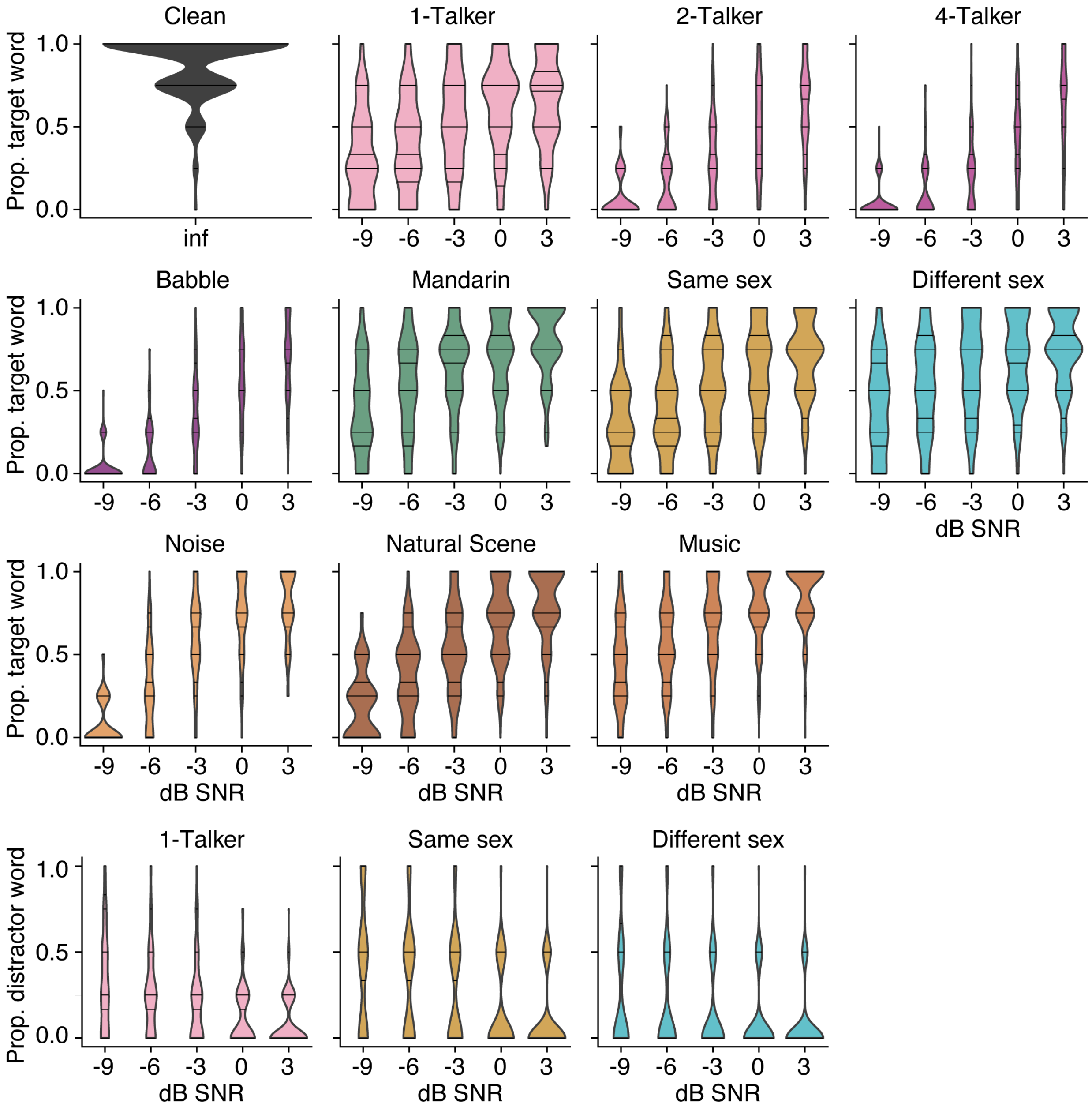

Supplementary Figure 1 | Data distributions for Experiment 1. Results of Experiment 1 plotted as distributions of the performance of individual participants (violin plots). This figure plots the same results as Figure 2a-e, but separates each stimulus condition into a separate panel so that the data distributions can be shown.

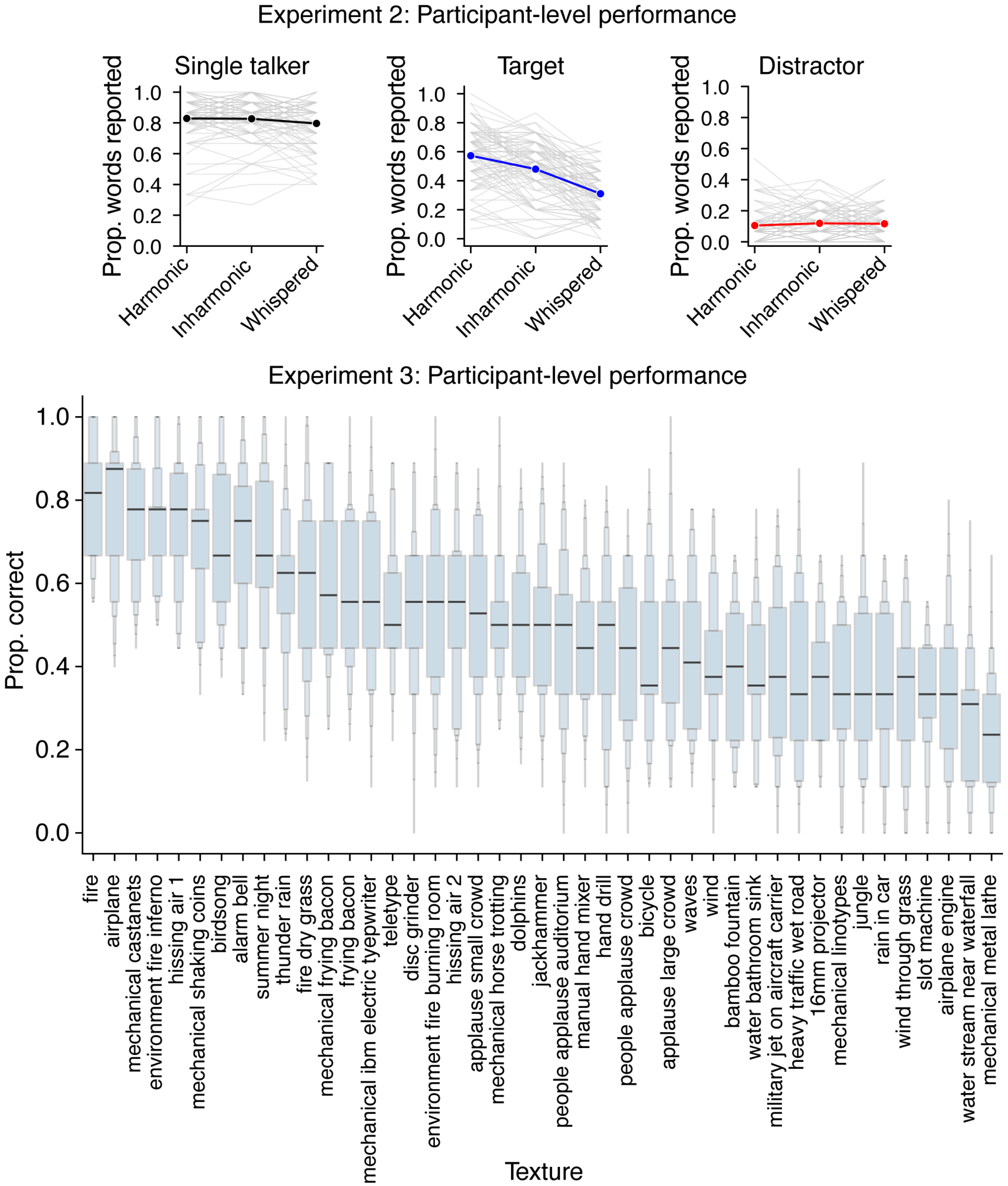

Supplementary Figure 2 | Data distributions for Experiments 2 and 3. Results of Experiments 2 and 3 plotted as distributions of the performance of individual participants. This figure plots the same results as Figure 2f&h, but separates each stimulus condition into a separate panel so that the data distributions can be shown. For Experiment 2 results (top), gray lines plot results for individual participants. For Experiment 3 results (bottom), data distributions are shown with letter-value plots.

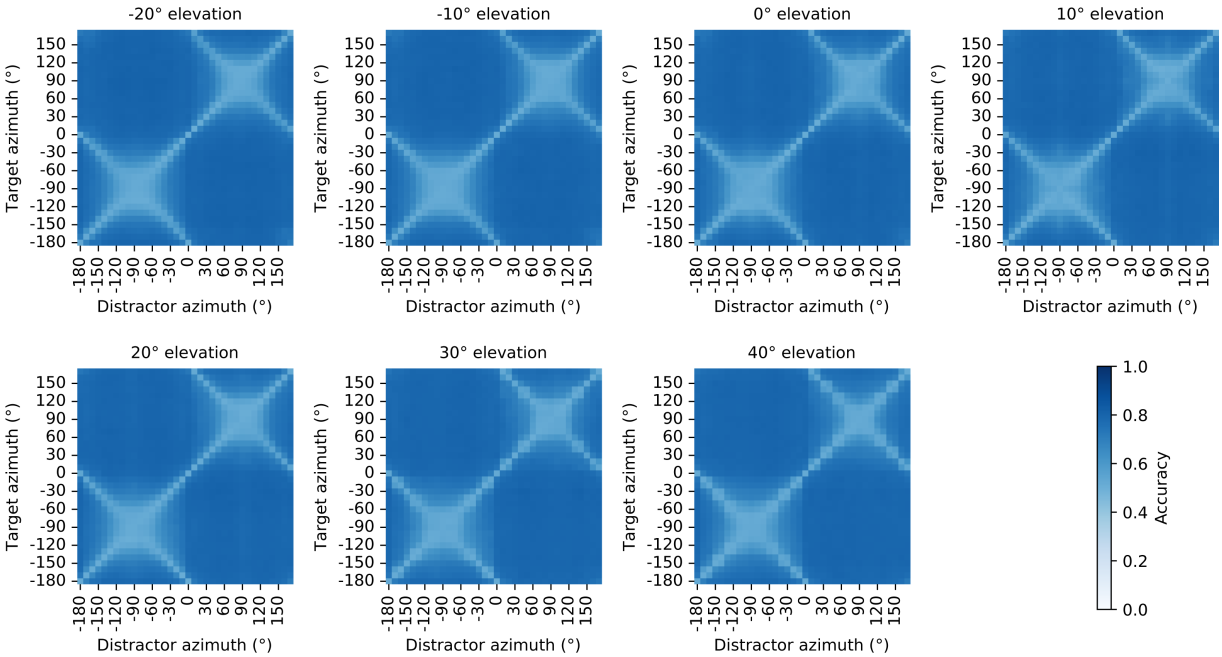

Supplementary Figure 3 | Model word recognition at all target-distractor azimuth pairings. Word recognition performance for the main feature-gain model (arch_v00 in Extended Data Table 1) as a function of target and distractor positions in azimuth. Each panel plots results for a different elevation, with target azimuth on the vertical axis and distractor azimuth on the horizontal axis (targets and distractors were at the same elevation). The plots differ from those in Figure 4 in plotting results for the full 360 degrees of azimuthal positions (for completeness). Because there was little effect of whether a source was in the front or back hemisphere, the plots in Figure 4 averaged the two hemispheres. The front-back symmetry yields the "X" structures evident in the plots. The broadening of spatial acuity as the target azimuth moved peripherally (i.e. approaching $\pm 90°$ from $0°$) was apparent at all elevations.

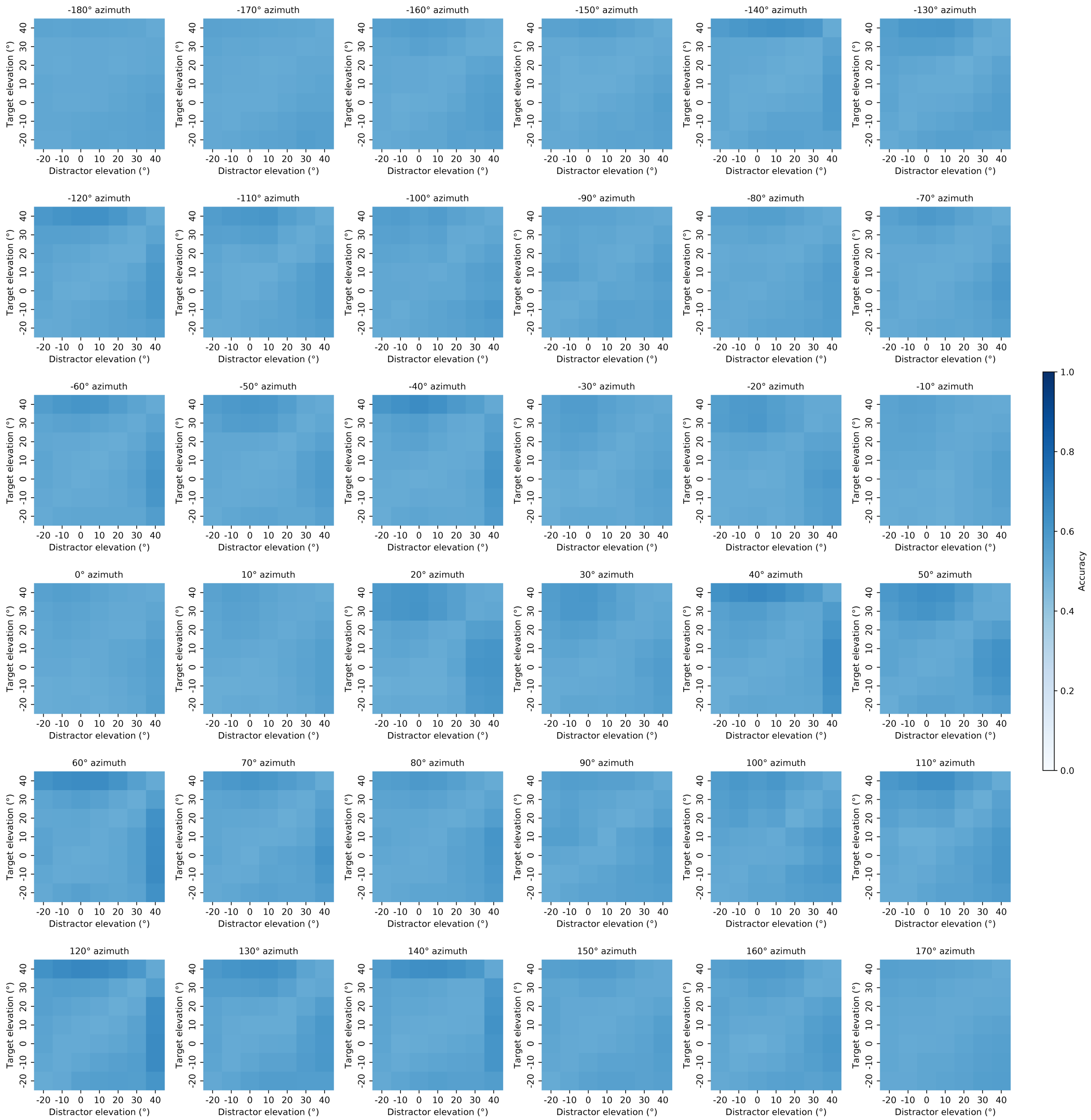

Supplementary Figure 4 | Model word recognition at all target-distractor elevation pairings. Word recognition performance for the main feature gain model (arch_v00 in Extended Data Table 1) as a function of target and distractor positions in elevation. Each panel plots results for a different azimuth, with target elevation on the vertical axis and distractor elevation on the horizontal axis (targets and distractors were at the same azimuth). Target-distractor offset in elevation produced little benefit regardless of azimuthal position.

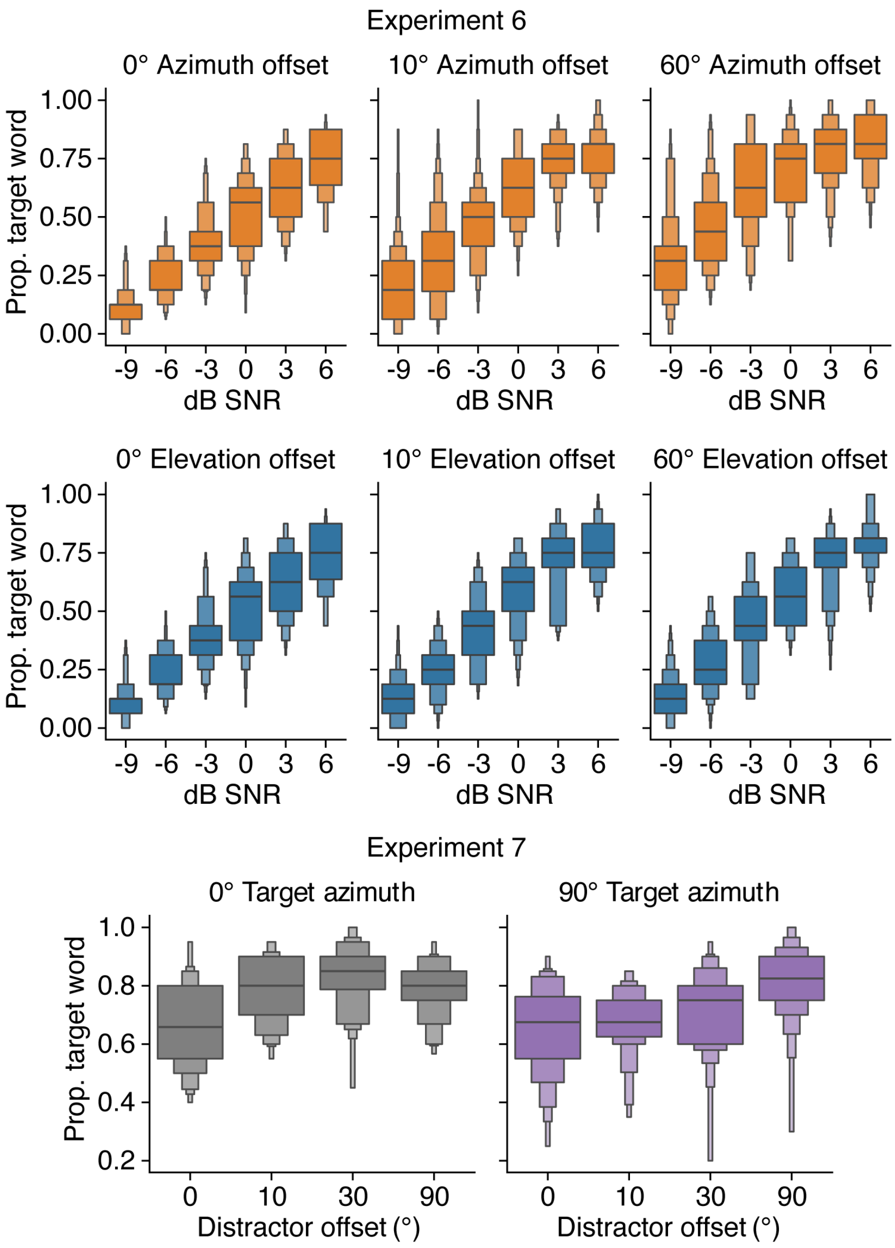

Supplementary Figure 5 | Data distributions for Experiments 6 and 7. Results of Experiments 6 and 7 plotted as distributions of the performance of individual participants. This figure plots the same results as Figure 4c&e, but separates each stimulus condition into a separate panel so that the data distributions can be shown (as letter-value plots).
