# Supplementary material for: Optimized feature gains explain and predict successes and failures of human selective listening
Source: Nat Hum Behav. 2026 Mar 13;10(5):937–59. doi: 10.1038/s41562-026-02414-7 (PMC13192276; doi:10.1038/s41562-026-02414-7)
Supplement: Supplementary file 2 — Reporting Summary [file 41562_2026_2414_MOESM2_ESM.pdf]

nature portfolio

Corresponding author(s): Ian Griffith and Josh H. McDermott

Last updated by author(s): Nov 17, 2025

Reporting Summary

Nature Portfolio wishes to improve the reproducibility of the work that we publish. This form provides structure for consistency and transparency in reporting. For further information on Nature Portfolio policies, see our Editorial Policies and the Editorial Policy Checklist.

Statistics

For all statistical analyses, confirm that the following items are present in the figure legend, table legend, main text, or Methods section.

n/a	Confirmed	
☐	☒	The exact sample size (*n*) for each experimental group/condition, given as a discrete number and unit of measurement
☐	☒	A statement on whether measurements were taken from distinct samples or whether the same sample was measured repeatedly
☐	☒	The statistical test(s) used AND whether they are one- or two-sided<br>*Only common tests should be described solely by name; describe more complex techniques in the Methods section.*
☒	☐	A description of all covariates tested
☒	☐	A description of any assumptions or corrections, such as tests of normality and adjustment for multiple comparisons
☐	☒	A full description of the statistical parameters including central tendency (e.g. means) or other basic estimates (e.g. regression coefficient) AND variation (e.g. standard deviation) or associated estimates of uncertainty (e.g. confidence intervals)
☐	☒	For null hypothesis testing, the test statistic (e.g. *F*, *t*, *r*) with confidence intervals, effect sizes, degrees of freedom and *P* value noted<br>*Give P values as exact values whenever suitable.*
☒	☐	For Bayesian analysis, information on the choice of priors and Markov chain Monte Carlo settings
☒	☐	For hierarchical and complex designs, identification of the appropriate level for tests and full reporting of outcomes
☐	☒	Estimates of effect sizes (e.g. Cohen's *d*, Pearson's *r*), indicating how they were calculated

Our web collection on statistics for biologists contains articles on many of the points above.

Software and code

Policy information about availability of computer code

Data collection	Modeling: computational modeling in Python version 3.11.5, Pytorch version 2.1.1, and Pytorch Lighting version 2.1.1. The code associated with this study is available at the project repository: https://github.com/mcdermottLab/auditory_attention<br><br>Human experiments: experiments used standard publicly available code packages; jsPsych 7.3 for web browser experiments posted on the Prolific platform, and Python version 3.12 for in-lab experiments.
Data analysis	Code made use of Python 3.11.5 packages: Pandas 2.1.3, Numpy 1.26.0, Matplotlib 3.8.0, Statsmodels 0.14.2, Seaborn 0.13.2, Scipy 1.11.3, Pingouin 0.5.5. The code associated with this study is available at the project repository: https://github.com/mcdermottLab/auditory_attention (Python environment to run code is included with the project repository).

For manuscripts utilizing custom algorithms or software that are central to the research but not yet described in published literature, software must be made available to editors and reviewers. We strongly encourage code deposition in a community repository (e.g. GitHub). See the Nature Portfolio guidelines for submitting code & software for further information.

Data

Policy information about availability of data

All manuscripts must include a data availability statement. This statement should provide the following information, where applicable:

- Accession codes, unique identifiers, or web links for publicly available datasets
- A description of any restrictions on data availability
- For clinical datasets or third party data, please ensure that the statement adheres to our policy

All model and human data are available at https://osf.io/wjzvu

Research involving human participants, their data, or biological material

Policy information about studies with human participants or human data. See also policy information about sex, gender (identity/presentation), and sexual orientation and race, ethnicity and racism.

Reporting on sex and gender	Gender was self-reported by study participants. A gender-based analysis was not performed as we were interested in the comparison between humans and computational models and did not investigate individual differences in human behavior.
Reporting on race, ethnicity, or other socially relevant groupings	Race and ethnicity information was collected via voluntary self-report but was not considered in any analyses as we were interested in the comparison between humans and computational models and did not investigate individual differences in human behavior.
Population characteristics	See "Behavioural & social sciences study design"
Recruitment	Online participants were recruited on the Prolific platform with a geographic filter set to exclude individuals outside of the United States. Participants were invited to perform "word recognition in noise" studies. In-person participants were recruited from the Cambridge, MA area via a mailing list.
Ethics oversight	The study was approved by the Committee on the Use of Humans as Experimental Subjects at MIT.

Note that full information on the approval of the study protocol must also be provided in the manuscript.

Field-specific reporting

Please select the one below that is the best fit for your research. If you are not sure, read the appropriate sections before making your selection.

☐ Life sciences ☒ Behavioural & social sciences ☐ Ecological, evolutionary & environmental sciences

For a reference copy of the document with all sections, see nature.com/documents/nr-reporting-summary-flat.pdf

Behavioural & social sciences study design

All studies must disclose on these points even when the disclosure is negative.

Study description	This quantitative study measured human abilities to recognize words in different conditions. Human data (from either online or in-person participants) was averaged across participants and compared to model performance.
Research sample	For the diotic word recognition experiments, online participants were used for convenience. We screened for self-reported normal hearing and did not screen for age or self-reported gender. Based on our previous experience running online experiments, this sample was representative of typical online participant cohorts. For the in-person experiments (in which sounds were presented from a speaker array), the participants were drawn from the Cambridge community. The sample is representative of normal hearing humans experienced with listening experiments.<br><br>Demographic information:<br>Experiment 1: 195 participants (98 female, 92 male, 4 non-binary, 1 no-report) between ages 18 and 71 (median 33) years.<br>Experiment 1b: 84 participants (43 female, 38 male, 3 non-binary) between ages 20 and 40 (median 32) years.<br>Experiment 2: 90 participants (43 female, 47 male) between ages 19 and 64 (median 34.5) years.<br>Experiment 3: Reproduced from Saddler et al., 2024; 47 participants (24 female, 23 male) between ages 23 and 59 (median 39) years.<br>Experiment 4: Reproduced from Byrne et al., (2023); 18 participants (11 female, 6 male, 1 non-specified; aged 18-40 years).<br>Experiment 5: Reproduced from Freyman et al., (1999); Originally reported as "56 young college students".<br>Experiment 6: 33 participants (22 female, 10 male) between ages 18 and 40 years (median age = 23).<br>Experiment 7: 28 participants (16 female, 12 male) between ages 19 and 39 years (median age = 26).
Sampling strategy	We used convenience sampling because we sought to characterize normal hearing listeners. We cannot exclude the possibility that self-selection biases could have resulted in a somewhat non-representative sample (because participants knew they were volunteering for a hearing experiment).

	Target sample sizes were determined via power analyses. For Experiment 1, we ran a pilot experiment with 95 participants, calculated the split-half reliability of the confusion rates across conditions (as this seemed likely to be the least reliable measure from the experiment), and estimated that a sample size of about 200 participants would yield a split-half reliability of .9. We performed a similar analysis for Experiment 2 but using a subset of 3 conditions from the same pilot experiment, and estimated that a sample size of about 80 participants would yield a split-half reliability of .9. For Experiment 6, we ran a pilot experiment with 9 participants, and estimated confidence intervals about the threshold for the co-located condition in samples of different sizes. We extrapolated that a sample size of about 30 participants would yield a confidence interval of 1 dB with 90% power. For Experiment 7, we sought to be able to detect a difference in performance for the 10-degree offset conditions with targets at either 0 or 90 degrees. We assumed a large effect size (d=0.8), yielding a target sample size of 19 to achieve 90% power. Experiments 3-5 used human data from previously published experiments.
Data collection	Online participants completed experiments in a web browser via surveys designed in jsPsych that played audio and recorded typed responses. In-person participants were played sounds from an array of loudspeakers and typed responses into a keypad. No one was present besides the participant and the researcher. The researcher was not blinded to the experimental condition or study hypothesis.
Timing	Data for Experiments 1 - 3 was collected online between May 2023 and Aug 2024. Data for Experiment 1b was collected in Sept 2025. Data for Experiments 6 and 7 were collected between December 2023 and February 2025.
Data exclusions	309 online participants were excluded from analyses for failing to pass the headphone check or performing poorly on independent catch trials. These were pre-established criteria meant to exclude participants that did not comply with online experiment instructions.
Non-participation	No in-person participant dropped out / declined participation. An unknown number of online participants on the Prolific platform dropped out, primarily due to technical difficulties (e.g., loss of internet connection or audio failing to play in the web browser). Participants could quit the experiments at any time without providing a reason.
Randomization	Participants were not allocated into experimental groups.

Reporting for specific materials, systems and methods

We require information from authors about some types of materials, experimental systems and methods used in many studies. Here, indicate whether each material, system or method listed is relevant to your study. If you are not sure if a list item applies to your research, read the appropriate section before selecting a response.

Materials & experimental systems

n/a	Involved in the study
☒	☐ Antibodies
☒	☐ Eukaryotic cell lines
☒	☐ Palaeontology and archaeology
☒	☐ Animals and other organisms
☒	☐ Clinical data
☒	☐ Dual use research of concern
☒	☐ Plants

Methods

n/a	Involved in the study
☒	☐ ChIP-seq
☒	☐ Flow cytometry
☒	☐ MRI-based neuroimaging

Plants

Seed stocks	*Report on the source of all seed stocks or other plant material used. If applicable, state the seed stock centre and catalogue number. If plant specimens were collected from the field, describe the collection location, date and sampling procedures.*
Novel plant genotypes	*Describe the methods by which all novel plant genotypes were produced. This includes those generated by transgenic approaches, gene editing, chemical/radiation-based mutagenesis and hybridization. For transgenic lines, describe the transformation method, the number of independent lines analyzed and the generation upon which experiments were performed. For gene-edited lines, describe the editor used, the endogenous sequence targeted for editing, the targeting guide RNA sequence (if applicable) and how the editor was applied.*
Authentication	*Describe any authentication procedures for each seed stock used or novel genotype generated. Describe any experiments used to assess the effect of a mutation and, where applicable, how potential secondary effects (e.g. second site T-DNA insertions, mosiacism, off-target gene editing) were examined.*
